# The impact of repetition mechanics on the adaptations resulting from strength-, hypertrophy- and cluster-type resistance training

**DOI:** 10.1007/s00421-016-3439-2

**Published:** 2016-07-29

**Authors:** G. Nicholson, T. Ispoglou, A. Bissas

**Affiliations:** Carnegie School of Sport, Institute for Sport, Physical Activity and Leisure, Leeds Beckett University, Fairfax Room 107, Headingley Campus, Leeds, LS6 3QT UK

**Keywords:** Rest interval, Cluster, Hypertrophy, Lactate, Muscle activity

## Abstract

**Purpose:**

The purpose of this study was to examine the acute and chronic training responses to strength-, hypertrophy- and cluster-type resistance training.

**Methods:**

Thirty-four trained males were assigned to a strength [STR: 4 × 6 repetitions, 85 % of one repetition maximum, (1RM), 900 s total rest], hypertrophy (HYP: 5 × 10 repetitions, 70 % 1RM, 360 s total rest), cluster 1 (*CL*-*1*: 4 × 6/1 repetitions, 85 % 1RM, 1400 s total rest), and cluster 2 (CL-2: 4 × 6/1 repetitions, 90 % 1RM, 1400 s total rest) regimens which were performed twice weekly for a 6-week period. Measurements were taken before, during and following the four workouts to investigate the acute training stimulus, whilst similar measurements were employed to examine the training effects before and after the intervention.

**Results:**

The improvements in 1RM strength were significantly greater for the STR (12.09 ± 2.75 %; *p* < 0.05, *d* = 1.106) and CL-2 (13.20 ± 2.18 %; *p* < 0.001, *d* = 0.816) regimens than the HYP regimen (8.13 ± 2.54 %, *d* = 0.453). In terms of the acute responses, the STR and CL-2 workouts resulted in greater time under tension (TUT) and impulse generation in individual repetitions than the HYP workout (*p* < 0.05). Furthermore, the STR (+3.65 ± 2.54 mmol/L^−1^) and HYP (+6.02 ± 2.97 mmol/L^−1^) workouts resulted in significantly greater elevations in blood lactate concentration (*p* < 0.001) than the CL-1 and CL-2 workouts.

**Conclusion:**

CL regimens produced similar strength improvements to STR regimens even when volume load was elevated (CL-2). The effectiveness of the STR and CL-2 regimens underlines the importance of high loads and impulse generation for strength development.

## Introduction

Resistance training (RT) is considered the primary modality when maximal strength development is the training goal (Baechle and Earle 2008) and the magnitude of adaptations resulting from an RT programme is known to be dependent on the specific combination of training-session components [load (mass lifted), volume, contraction velocity] in addition to other possible confounding variables (e.g. nutritional status). From a scientific perspective, evidence suggests that different combinations of training-session components result in different external mechanical stimuli (e.g. time under tension, Crewther et al. 2005) and that these stimuli interact to produce different acute physiological (e.g. metabolic) responses (Crewther et al. 2006). When applied over a period of time, these mechanical and physiological stimuli influence the type and magnitude of training adaptations (Kraemer et al. 1990; Goto et al. 2005).

Although ‘light’ and ‘maximal’ RT workouts may not be mutually exclusive (Burd et al. 2012), coaches typically distinguish between strength- (STR), hypertrophy- (HYP) and endurance-type RT. Based on the current terminology, it would appear that STR regimens involving high loads [≥85 % of one repetition maximum (1RM)], low volumes (2–6 sets, ≤6 repetitions) and long inter-set rest intervals (3–5 min) represent the optimal method of improving maximal strength (Baechle and Earle 2008) with the current recommendations reflecting the prevailing view (Carpinelli 2008) that high loads (≥85 % 1RM) need to be utilised to maximise strength development. The main basis for the prescription of high loads is based on the size principle of motor unit recruitment (Henneman 1957) and research which supports the need for progressively higher forces to enable the recruitment of the higher threshold motor units (Gordon et al. 2004). Although the prescription of high loads during STR regimens would appear to be based on a sound scientific basis, studies exist which suggest that higher threshold motor units can also be recruited in the absence of high loads when blood flow restriction is present (Moore et al. 2004) or in the latter stages of submaximal exercise when fatigue levels are higher (Houtman et al. 2003). These observations may explain why the longitudinal evidence in support of STR regimens is far from unequivocal (Carpinelli 2008).

Considering the conflicting literature, it is perhaps not surprising that there is an emerging body of evidence which questions the importance of high loads for maximal strength development with metabolic stress and the associated muscle ischemia also being put forward as key stimuli (Goto et al. 2005). Despite the fact that metabolite accumulation as indicated by blood lactate and hydrogen ion accumulation is known to be maximised following HYP regimens that involve high volumes (3–6 sets, 8–12 repetitions), moderate loads (67–80 % 1RM) and short rest intervals (30–90 s) (Nicholson et al. 2014), the need for metabolite accumulation has often been argued based on the effectiveness of other training practices since low-load RT workouts involving blood flow restriction have shown to be effective for enhancing strength and muscle hypertrophy (Takarada et al. 2002). Although it has been suggested that metabolic stress may be an important driver of the intracellular protein synthesis signalling pathway (Schoenfeld 2010), recent research suggests that regimens involving high metabolic stress may not be as effective for enhancing the neural contributions to strength gains (Moore et al. 2004; Sakamaki et al. 2012). Furthermore, the role(s) of metabolic stress and circulating hormones in the hypertrophic response is not fully understood (West et al. 2009) and evidence actually exists which suggests that the metabolic stimulus is unlikely to be the primary and only stimulus for strength development (Yasuda et al. 2011). Instead, evidence suggests that elevated metabolic responses in combination with high loads may have an additive effect on the development of maximal strength (Goto et al. 2005). Despite the scientific underpinning, a poor understanding exists regarding the magnitude of metabolic stress required to optimise strength adaptations.

In addition to research which supports the need for metabolic stress, evidence exists to suggest that high-threshold motor unit recruitment is also possible when the acceleration of a load is optimised (Linnamo et al. 2000). Whilst it is generally agreed that elevating lifting velocity through the use of low loads will result in smaller strength improvements than high-load training (Kaneko et al. 1983), recent research presents the possibility that lifting velocity may be elevated without sacrificing the load lifted. More specifically, the inclusion of short (30–90 s) rest intervals in between small groups of repetitions has been termed ‘cluster set’ training (CL). Compared to traditional STR workouts, CL workouts have been shown to allow greater velocities and power outputs to be maintained over multiple sets (Haff et al. 2003) albeit with a lower level of metabolic stress (Goto et al. 2005). Whilst the ability to optimise repetition kinematics without sacrificing training load represents an exciting proposition, a poor understanding exists concerning the relative importance of these kinematic responses for maximal strength development (Crewther et al. 2005). Furthermore, it has been theorised that kinetic responses such as force, time under tension and the product of the two (impulse) may play a key role in the strength training stimulus (Crewther et al. 2005; Neils et al. 2005). Whilst these theories appear logical, there is a lack of research that has examined the relative importance of repetition mechanics, metabolic stress and chronic adaptations within the same investigation. It is, therefore, not surprising that a lack of consensus exists regarding the best ways to utilise the CL concept.

At present, studies exist both for (Oliver et al. 2013) and against (Goto et al. 2005) the effectiveness of CL when the goal is maximal strength development. A major limitation of previous CL studies is that they have failed to consider that more frequent rest allows higher loads to be utilised without sacrificing repetition number (Iglesias et al. 2010). Although the ability to enhance volume load in this manner may enhance motor unit recruitment, it is at odds with the so-called ‘repetition maximum continuum’ (Baechle and Earle 2008). Despite evidence which supports greater neural adaptations following explosive (Linnamo et al. 2000) and heavy (Hakkinen et al. 1987) RT, there is a lack of information regarding the neural responses to CL regimens (Iglesias-Soler et al. 2015). Research which examines the neural responses to CL training will assist practitioners in designing RT programmes for sports in which relative strength is important.

The aim of this study was to compare the acute (metabolic and mechanical) and chronic responses to STR, HYP, and two novel CL regimens involving the back-squat exercise. This approach was intended to answer key questions regarding the magnitude and type (i.e. neural) of adaptations resulting from workouts which set out to emphasise contrasting mechanical and metabolic responses. We hypothesised that the CL regimens would optimise the acute kinematic and kinetic responses with an attenuated metabolic response and that a CL regimen which permits a higher load would result in the largest increases in strength and muscle activity.

## Methods

### Subjects and experimental design

The present study consisted of two separate investigations. Forty-six male subjects (age: 21.76 ± 2.60 years; height: 178.0 ± 6.3 cm; body mass: 81.14 ± 8.83 kg; 1RM: body mass ratio: 1.6 ± 0.3) volunteered to participate in both parts of the study. The subjects were chosen due to their experience in structured strength training (minimum 12 months) and their proficiency in the back-squat exercise. During familiarisation, if subjects were unable to complete multiple repetitions (>8) of the parallel back squat with a weight equal to their own body mass on the bar they were excluded from the study. All subjects were not taking medication or any other nutritional supplements (e.g. creatine) known to affect energy metabolism or physical performance. The Faculty’s Research Ethics Committee approved the details of the study and all subjects gave written informed consent to indicate their voluntary participation.

**Fig. 1:**
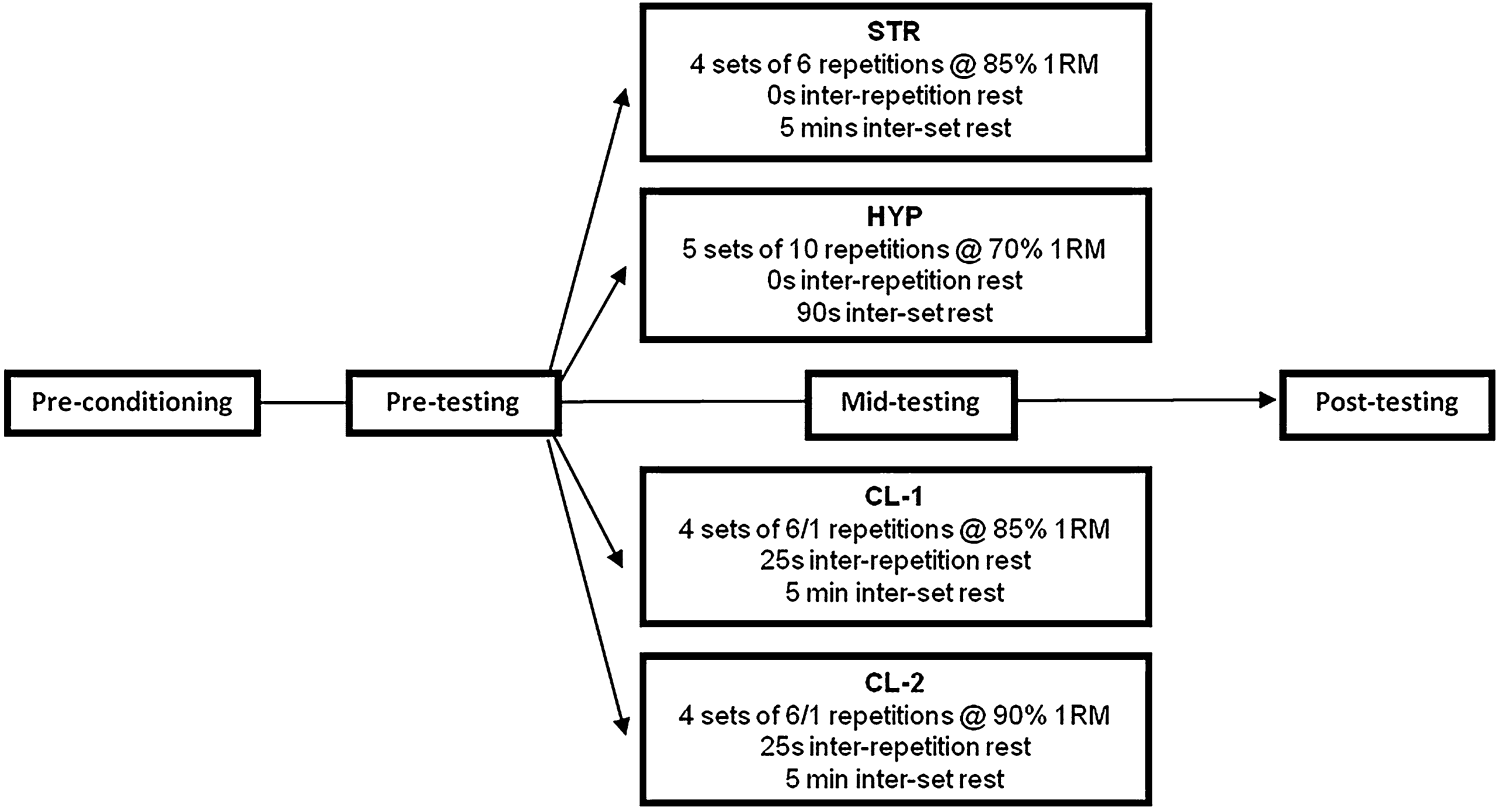
Schematic representation of the experimental groups and design. *6/1* denotes six repetitions performed as singles

Following familiarisation and a standardised two-week pre-conditioning period, subjects were matched according to 1RM strength in the back-squat exercise and then assigned to either a strength- (STR; *n* = *11*) or hypertrophy-type (HYP; *n* = *12*) regimen, a cluster-type (CL) regimen involving greater total resting time (CL-1; *n* = *12*) or a CL regimen involving greater total rest and volume load (CL-2; *n* = *11*). The primary investigation then examined the chronic effects of these back-squat workouts performed twice weekly for a 6-week period. Specifically, subjects were tested for dynamic, isometric and isokinetic strength, sEMG activity and power performance during 1 testing session at pre-, mid- and post-training (Fig. 1). The secondary investigation examined the acute effects of the experimental workouts on blood lactate (BL) concentration and repetition quality during one visit to the laboratory.

### Study of chronic responses

#### Subjects and training regimen

Of the 46 subjects that were recruited for the study, 34 subjects completed all training sessions and tests. Prior to the commencement of the experimental training period, all subjects completed a standardised 2-week pre-conditioning period involving three weekly sessions and exercises of the upper and lower body and major muscles of the trunk and abdomen; this was included to ensure that the sample was as homogenous as possible. Following pre-conditioning, baseline 1RM assessments and assignment to the four training groups, subjects performed two back-squat workouts per week for a six-week period. Although RT programmes typically involve whole-body regimens with higher weekly volumes, our study included only one exercise as has been done previously (Cormie et al. 2010). Subjects were instructed to lower the load at a constant velocity but complete the concentric phase of each repetition as fast as possible. Training sessions were preceded by a standardised warm-up involving 5 min of cycling on a stationary ergometer, dynamic mobility exercises and submaximal sets of the back squat at 50 and 75 % of the prescribed training intensity.

The volume, loads and rest intervals involved in each regimen are shown in Fig. 1. Subjects performed their designated back-squat workout whilst being supervised by a member of the research team. Subjects were instructed not to perform any additional resistance training other than one upper body training session (performed once weekly) that was identical (load, volume, rest interval duration, exercises) for all four training groups. To account for any possible confounding variables, subjects completed a physical activity record in which they recorded the type, duration and perceived exertion of all sporting activities. Ratings of perceived exertion (RPE) were also recorded at the end of all resistance training sessions using a 10-point scale (Borg 1982) to provide an indication of the demands associated with the experimental workouts. In addition, ratings of perceived soreness (RPS) were completed 24 h after each training session using a 10-point scale to gain an idea of the pain and discomfort associated with each training session. Subjects were asked to maintain their habitual diet throughout the training period and kept a diet diary for a period of three continuous days at the mid-point of the study. The diet diaries were analysed using NetWISP dietary analysis software (Version 3.0; Tinuviel Software, Warrington, UK) to test for any possible differences in energy and macronutrient intake between the groups.

#### Measurements of muscular strength

Dynamic strength was evaluated as 1RM for the back-squat exercise and using an isokinetic knee extension/flexion movement. Maximal isometric strength was also measured for the back-squat exercise. Procedures to measure 1RM strength were identical to those previously described (Campos et al. 2002) and briefly involved a series of submaximal warm-up sets followed by five maximal lifting attempts until each subject’s 1RM was identified. Periods of rest (approximately 4–5 min) were permitted between trials in an attempt to maintain maximal performance. Successful attempts required subjects to descend to the point where the tops of the thighs were parallel to the floor and squat depth was visually assessed by the same experienced researcher. All 1RM testing sessions took place in the same exercise laboratory using a customised power rack with adjusted safety stoppers and were performed at least 72 h prior to the experimental sessions.

The maximal isometric back squat was performed pre-, mid- and post-training to measure changes in subjects’ bilateral strength. The subjects were familiarised with the testing procedures and set up on two separate occasions prior to the start of the experimental training period. On each occasion, subjects performed three maximum trials interspersed with 3-min rest intervals. Participants were instructed to push as hard and as fast as possible and to maintain this level of excitation for 4 s. Trials with an observable unloading in the vertical ground reaction force–time curve immediately prior to the commencement of contraction were repeated (Cormie et al. 2010). Subjects performed the isometric back squat using a modified squat rack positioned over a force platform (1000 Hz; Kistler, Winterthur). A knee angle of 100^°^ was selected based on previous reliability observations (Nicholson and Bissas 2015) and was verified at the start of each data collection section using a clinical goniometer. Isometric back-squat performance was analysed using Bioware software (version 5.0; Kistler, Winterthur). This required the identification of Maximal Bilateral Isometric Force (MBIF) and several measures of rate of force development (RFD) from the resultant force–time curve. Measures of RFD included the force produced at various time points [e.g. 0–100 milliseconds (ms)] and the time taken to achieve relative proportions of MBIF (e.g. 25, 50 %). The above variables were reported for the trial with the highest MBIF at pre- and post-training.

The isokinetic test was used as a dynamic test to detect any joint velocity-specific strength changes following the training period. A Cybex norm dynamometer was used to administer a knee extension/flexion test (concentric–concentric) protocol at a slow (30^°^/s) and medium (180^°^/s) angular velocity. Subjects were seated, hip angle was standardised at 90^°^ and subjects were instructed to perform all trials with the ankle in a dorsi-flexed position. The shank was attached to the dynamometer lever arm above the medial malleolus and the rotational axis of the dynamometer was visually aligned with the lateral femoral epicondyle under active conditions. Stabilisation was achieved via strapping of the shoulders, torso and distal thigh along with immobilisation of the contralateral limb. Subjects completed 1 set of 5 maximal repetitions at each velocity with 3-min rest between each set. Peak extensor and flexor torque was identified at each angular velocity and the highest torque of the five maximal trials was reported pre- and post-training. Additional analysis included the calculation of reciprocal muscle group ratios at each angular velocity (peak flexor torque/peak extensor torque) and the identification of the angle at which peak torque occurred.

#### Measurements of electromyographic activity

Concurrent surface electromyographic activity (sEMG) was recorded during the isometric back-squat assessment for the vastus medialis, rectus femoris and gluteus maximus. Skin preparation involved shaving and cleaning the skin surface with alcohol swabs. Two active bipolar electrodes (Delysys Inc., Boston, MA) with a 10-mm fixed inter-electrode distance were placed on each muscle and a reference electrode was placed on the lateral condyle of the femur. Electrodes were placed on the muscle belly parallel to the underlying muscle fibres relative to key anatomical landmarks as recommended by Hermens et al. (1999). A telemetry unit (Myomonitor IV; Delysys Inc., Boston, MA, USA) was used to collect the data at 1000 Hz (CMRR >80Db, gain = 1000, input impedance = 10 MΩ). To reduce movement artefact, wires connecting the electrodes to the unit were held in place by tubular net bandages. The raw sEMG signals were analysed for a single trial at pre-, mid- and post-training based on the trial which elicited MBIF at each time point. The sEMG data were first filtered using a band pass filter (cutoff frequencies: 20–450 Hz) and the mean amplitude [root mean square (RMS)] and median frequency (MF) was then obtained for a 0.5-s period corresponding to the time point of MBIF (identified from the synchronised force/torque-time curves). Mean amplitude was expressed in absolute units (microvolts) at pre-, mid- and post-training for each of the muscles analysed.

#### Measurements of muscular power

Vertical countermovement jumps were performed on a force platform with external loads of 0, 20 and 40 kg to evaluate changes in stretch-shortening cycle function under a number of different loading conditions. On each occasion subjects performed three maximal trials of each jump assessment on the force platform interspersed with 3-min rest intervals. After starting from an upright position, subjects were instructed to descend to a self-selected depth and then jump for maximum vertical displacement (Hansen et al. 2011). The 20 and 40 kg CMJ trials were performed with a 20 kg Olympic barbell position across the shoulders immediately above C7 (Cormie et al. 2010). The analysis of the jumps initially involved the calculation of jump height via the flight time (time subjects spent airborne in each jump) method (9.81 × flight time^2^). In addition to the calculation of jump height, a number of common (e.g. peak power) and more in-depth kinetic (e.g. concentric impulse), temporal (e.g. duration of concentric/eccentric phases) and slope-related (e.g. rate of power development) variables were calculated. The additional variables were calculated for the best of three maximal trials (identified as the highest jump) at pre- and post-training.

### Study of acute responses

#### Subjects and acute resistance training bouts

The 46 subjects that were initially recruited had their acute responses to a single training session examined in a visit to the laboratory. Subjects in the STR, HYP, CL-1 and CL-2 groups performed the same workouts as those used to examine the chronic responses. Finger-tip blood samples were carried out before and after the training sessions to provide information regarding the level of metabolic stress resulting from each session. Prior to each testing session, subjects underwent 2 h fasting and were advised to rest for 24 h beforehand. The same standardised warm-up (described above) was performed prior to each session and all workouts were supervised by the same member of the research team.

#### Blood sampling and analyses

Finger-tip blood samples were taken immediately pre- and post-workout to examine the metabolic stress induced by the experimental workouts. All samples were obtained with subjects in a seated position and each subject’s finger was cleaned using Alcotip swabs prior to each sample before being pierced using a sterile lancet (Kendall, Manolet Monoject, UK). The first drop of blood was wiped away to avoid contaminating the sample and the subject’s blood was then collected in a 25-µL microvette tube by ‘milking’ the proximal end of the index finger. The samples were immediately analysed for blood lactate (BL) concentration using the YSI 2300 stat plus (Yellow Springs, USA). This device was calibrated prior to each testing session using assays of a known concentration.

#### Measurement of mechanical characteristics

To capture changes in repetition kinetic and kinematics during the experimental workouts, subjects performed all their repetitions on a force platform (Kistler 9281EA; 1000 Hz). This enabled the collection of ground reaction force data in the medio-lateral, anterior–posterior and vertical directions. Following filtering with a low-pass Butterworth filter at a cutoff frequency of 10 Hz, a range of variables was calculated to represent the changes in repetition mechanics within and between sets. The variables included average concentric vertical force (N/kg), peak anterior–posterior force (N/kg), average concentric vertical impulse (Ns/kg), concentric time under tension (s), average concentric vertical velocity (m/s), and average concentric vertical power (W/kg). These variables were calculated for the first, middle and last repetition of each set. The percentage change from the first to the last repetition was calculated for each set. Furthermore, the values for each repetition (first, middle and last repetition of each set) were combined together over the course of the workouts (sets 1–4/5) to produce a mean response for each individual repetition over the course of each workout.

### Statistical analysis

Means and standard deviations of the experimental variables were initially calculated. Levene’s test was used to check for equality of variances whilst distribution parameters were used to determine the appropriateness of parametric tests. The acute and chronic results were primarily analysed with a 4 × 3 (group × time) mixed factorial analysis of variance (ANOVA) apart from the repetition quality data which were initially analysed with a 4 × 4 × 3 (group × set × repetition) mixed ANOVA. In the event of a significant interaction, multiple comparisons were performed with Bonferroni adjustment. A one-way ANOVA was also performed on the absolute and percentage changes with subsequent post hoc analysis (using Tukey’s HSD) to identify any differences in the magnitude of the acute or chronic changes between the training groups. Mean effect sizes (ES, Cohen’s d) were calculated using the mean pre- and post-training values to compare the improvements among the experimental groups along with 95 % confidence intervals of the difference. A statistical package (SPSS version 19.0, IBM Inc., Chicago, Illinois, USA) was used to perform all statistical analyses and statistical significance was set at *p* < 0.05 for all of the tests performed.

## Results

### Chronic responses

#### Changes in muscular strength

**Fig. 2:**
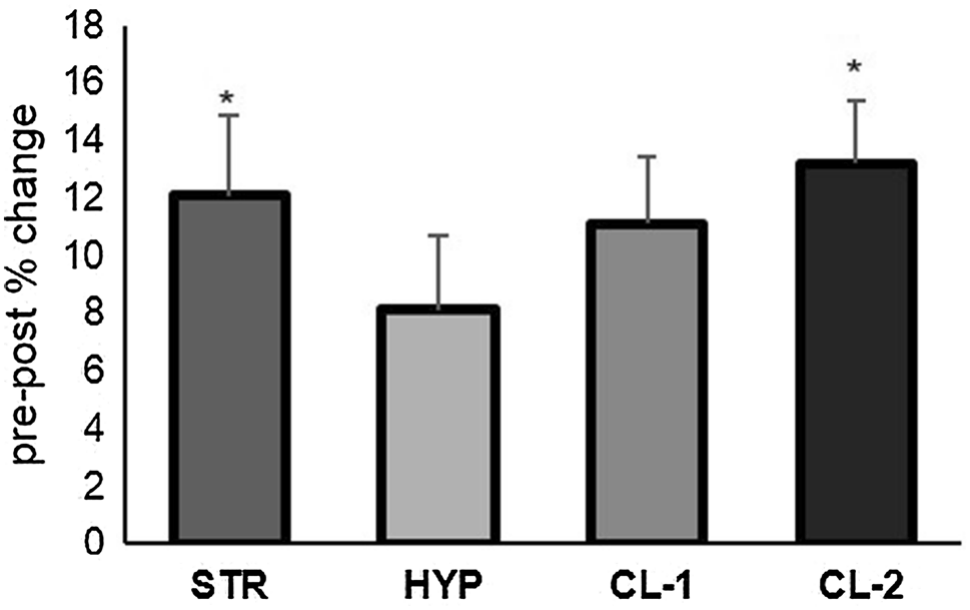
Pre- to post-training percentage changes in 1RM back-squat strength. *Asterisks* significant difference from the HYP regimen (*p* < 0.05)

No significant differences were observed between groups at pre-training for 1RM, MBIF or isokinetic peak torque at any of the measurement velocities. Changes in 1RM at post-training are shown in Table 1. All four training groups showed significant increases (*p* = 0.000) in 1RM at mid- and post-training with the post-training increases in normalised 1RM ranging between 8 and 13 %. Figure 2 shows the differences in the normalised 1RM improvements for four training groups. In terms of effect size, the STR regimens demonstrated a large effect compared to those reported for the HYP regimen. Whilst the effect sizes for the CL-1 and CL-2 regimens were largely similar when calculated for the normalised 1RM changes, the CL-2 demonstrated a larger effect than the CL-1 regimen for the absolute 1RM changes.

**Table 1:** Changes in 1RM back squat strength during and following the training period

	Pre	Post	Pre-post change (kg)	ES	95 % CI
Dynamic strength					
1RM (kg)					
STR	120.56 ± 13.96	135.83 ± 13.64*	15.28 ± 1.95^HYP^	1.106	13.34–16.66
HYP	124.29 ± 24.01	135.36 ± 24.81 *	11.07 ± 2.44	0.453	8.14–13.29
CL-1	134.72 ± 21.88	150.56 ± 23.78*	15.83 ± 3.54	0.693	15.75–19.80
CL-2	121.39 ± 21.36	138.61 ± 20.85*	17.22 ± 2.32^HYP^	0.816	12.71–18.40
1RM (kg/BM)					
STR	1.55 ± 0.15	1.73 ± 0.16*	0.19 ± 0.04^HYP^	1.161	0.15–0.21
HYP	1.64 ± 0.34	1.70 ± 0.30*	0.12 ± 0.04	0.187	0.08–0.20
CL-1	1.60 ± 0.28	1.77 ± 0.29*	0.17 ± 0.04	0.588	0.13–0.20
CL-2	1.59 ± 0.33	1.79 ± 0.35*	0.21 ± 0.03^HYP^	0.596	0.19–0.24

All four training groups showed mean increases in MBIF measured during the isometric back-squat protocol which were accompanied by a significant main effect of time (*p* = 0.000). In terms of influence of the training, there was no significant interaction between the two factors (time × group) and no significant differences in the magnitude of the improvements. Similar to MBIF, all four groups also showed mean increases in isometric RFD at post-training. The increases in RFD were characterised by reductions in the time taken to achieve various relative (e.g. 25 %) levels of MBIF and increases in the slope of the force–time curve during the first 100–200 ms of contraction. Although the mean increases in RFD were accompanied by significant main effects of time (*p* < 0.01), there were no differences in the magnitude of the RFD improvements between the training groups.

Table 2 shows the isokinetic performance for the knee extensors and flexors at pre- and post-training when measured at 30^°^/s and 180^°^/s. All four groups showed mean increases in peak extensor and flexor torque at both angular velocities which were accompanied by significant main effects of time (*p* = 0.000). Interestingly, a significant interaction was observed for the peak extensor values measured at 30^°^/s (*p* = 0.035) with further analysis revealing significant improvements for the STR (*p* = 0.001), CL-1 (*p* = 0.035) and CL-2 groups (*p* = 0.000) with the improvements for the CL-2 group (17.12 ± 16.00 %) being significantly greater than those observed for the HYP (*p* = 0.020, 5.22 ± 8.35 %) and CL-1 (*p* = 0.049, 4.42 ± 5.09 %) group. No significant changes were observed in the angle at which peak torque occurred for any of the training groups at either measurement velocity.

**Table 2:** Changes in normalised isokinetic strength after the training period

	Pre-post change (Nm/kg)	ES	95 % CI
Isokinetic strength			
PT Ext 30^°^/s			
STR	0.21 ± 0.13	0.40	0.09–0.36
HYP	0.13 ± 0.23	0.28	−0.04 to 0.31
CL-1	0.13 ± 0.15	0.34	−0.29 to 0.21
CL-2	0.45 ± 0.39	1.60	0.15–0.77
PT Flex 30^°^/s			
STR	0.11 ± 0.15	0.49	−0.12 to 0.24
HYP	0.19 ± 0.17	0.65	−0.51 to 0.49
CL-1	0.08 ± 0.16	0.24	−0.03 to 0.21
CL-2	0.19 ± 0.11	0.75	0.09–0.26
PT Ext 180^°^/s			
STR	0.15 ± 0.15	0.43	0.01–0.28
HYP	0.23 ± 0.11	0.92	0.04–0.36
CL-1	0.19 ± 0.17	0.30	0.04–0.32
CL-2	0.18 ± 0.20	0.69	0.04–0.36
PT Ext 180^°^/s			
STR	0.13 ± 0.16	0.86	−0.01 to 0.24
HYP	0.14 ± 0.12	0.51	0.05–0.23
CL-1	0.14 ± 0.08	0.77	0.10–0.21
CL-2	0.19 ± 0.13	0.61	0.10–0.32

*PT* peak torque

#### Changes in muscle activity

The increases in MBIF were accompanied by mean post-training increases in RMS activity for the rectus femoris (34–59 %), vastus medialis (8–22 %) and gluteus maximus (8–22 %) for all of the groups which in some instances were accompanied by post-training increases in MF (≤18 %). Whilst significant main effects of time were observed for the RMS (*p* < 0.01) and MF (*p* < 0.05) values, there were no differences in the magnitude of the changes between groups.

#### Changes in vertical jump performance

There were no significant differences between groups for any jump performance variable at baseline. Table 3 shows that all four groups showed mean post-training increases in jump height (5–13 %) and power output (2–6 %) at all external loads which were accompanied by significant main effects of time (*p* < 0.001). Importantly, there were no differences in the magnitude of the jump height or power improvements between groups.

**Table 3:** Pre- and post-training measures of jump performance when measured at loads of 0, 20 and 40 kg

	STR		HYP		CL-1		CL-2	
	Pre	Post	Pre	Post	Pre	Post	Pre	Post
Vertical jump performance								
Jump height (m)								
0 kg	0.39 ± 0.07	0.41 ± 0.07	0.35 ± 0.06	0.38 ± 0.06	0.34 ± 0.05	0.36 ± 0.05	0.34 ± 0.05	0.36 ± 0.04
20 kg	0.26 ± 0.06	0.28 ± 0.05	0.23 ± 0.05	0.26 ± 0.06	0.24 ± 0.04	0.25 ± 0.04	0.22 ± 0.03	0.24 ± 0.03
40 kg	0.19 ± 0.05	0.20 ± 0.05	0.17 ± 0.06	0.19 ± 0.05	0.18 ± 0.04	0.19 ± 0.04	0.17 ± 0.03	0.19 ± 0.03
Peak power (W/kg)								
0 kg	56.27 ± 8.80	58.90 ± 9.47	51.79 ± 7.93	53.86 ± 7.64	50.07 ± 5.24	52.70 ± 4.48	51.15 ± 6.74	51.96 ± 5.45
20 kg	53.14 ± 10.47	54.55 ± 9.77	48.29 ± 6.36	49.89 ± 7.32	48.81 ± 4.95	50.08 ± 4.17	46.32 ± 4.65	48.22 ± 4.74
40 kg	52.06 ± 8.42	54.43 ± 8.28	47.61 ± 6.59	50.04 ± 8.39	49.15 ± 5.40	51.47 ± 3.71	46.17 ± 4.31	48.63 ± 3.81
Peak force (N/kg)								
0 kg	25.68 ± 2.67	24.54 ± 1.98	24.11 ± 2.00	24.35 ± 1.69	23.24 ± 1.67	23.63 ± 2.19	25.08 ± 1.55	24.29 ± 1.99
20 kg	26.07 ± 2.36	25.03 ± 1.71	24.74 ± 1.66	25.55 ± 1.04	24.50 ± 1.76	24.97 ± 1.00	23.90 ± 0.83	24.60 ± 1.42
40 kg	27.81 ± 1.66	27.90 ± 1.98	27.10 ± 2.03	28.01 ± 2.03	27.00 ± 1.94	27.52 ± 0.86	25.75 ± 1.39	26.55 ± 1.18
Displacement (m)								
0 kg	0.45 ± 0.06	0.47 ± 0.06	0.44 ± 0.06	0.47 ± 0.05	0.43 ± 0.06	0.44 ± 0.05	0.44 ± 0.07	0.46 ± 0.07
20 kg	0.43 ± 0.04	0.42 ± 0.04	0.44 ± 0.07	0.48 ± 0.06*	0.43 ± 0.07	0.42 ± 0.06	0.43 ± 0.03	0.46 ± 0.06
40 kg	0.42 ± 0.05	0.46 ± 0.05	0.41 ± 0.07	0.42 ± 0.05	0.41 ± 0.05	0.42 ± 0.07	0.42 ± 0.05	0.43 ± 0.05
Work (J)								
0 kg	614.71 ± 97.49	656.38 ± 129.41	612.77 ± 113.75	653.19 ± 123.33	669.82 ± 90.86	689.21 ± 76.88	596.23 ± 99.70	630.92 ± 121.36
20 kg	642.45 ± 76.16	667.58 ± 76.76	673.68 ± 143.62	749.79 ± 130.35*	630.71 ± 220.72	691.24 ± 253.64*	650.18 ± 130.87	651.75 ± 101.03
40 kg	705.17 ± 75.92	755.19 ± 82.12	704.01 ± 151.67	736.11 ± 135.33	745.88 ± 70.93	785.57 ± 115.59	690.49 ± 110.11	716.64 ± 107.37

* Significant difference from pre-training (*p* < 0.05)

In terms of the mechanisms underlying the improvements in jump height, increases in peak velocity were observed at all external loads which were accompanied by significant main effects of time (*p* < 0.001), however, a significant main effect of time (*p* = 0.035) was only observed for peak force when jumps were performed with an external load of 40 kg. Interestingly, the HYP (12.22 ± 9.07 %) and CL-1 (8.79 ± 6.20 %) groups were the only regimens that induced significant increases (*p* < 0.05) in work done when jumps were performed at 20 kg. Furthermore, the HYP group showed a significant increase in displacement (12.68 ± 15.21 %, *p* = 0.045), a significant increase in the duration of the concentric phase (10.90 ± 10.64 %, *p* < 0.001) and a significant reduction in the duration of the eccentric phase (−5.29 ± 5.10, *p* < 0.001) when the jumps were performed with an external load of 20 kg.

#### Perceived exertion and muscle soreness

The mean RPE for the training period was significantly greater (*p* = 0.042) for the HYP group (7.62 ± 1.17) when compared to the CL-1 group (5.99 ± 1.07). The RPE for the HYP group remained consistently higher throughout the training period and was significantly greater than that reported for the CL-1 group during sessions 1 (*p* = 0.020), 3 (*p* = 0.047) and 7 (*p* = 0.031) of the training period. The mean RPS ranged from 1.91 to 3.61 which corresponded to slight/more than slight pain on the scale. There were no statistical differences in RPS values at any point during the training intervention.

#### Physical activity and nutritional intake

The total energy intake, macronutrient and fluid intake were similar between the four training groups over the 3-day sample period with no significant differences being observed between groups. The percentage macronutrient contribution to the total energy intake was also similar with no significant between-group differences being observed. Furthermore, there were no significant changes in body mass from pre- to post-training for the STR (0.70 ± 0.86 %), HYP (0.94 ± 1.70 %), CL-1 (0.89 ± 1.94 %) or CL-2 (0.82 ± 1.01 %) groups.

In addition to the activity performed as part of the experimental training period, the time spent performing low-, moderate-, high- and maximal-intensity physical activity was very similar between the training groups with no significant differences being observed.

#### Load and volume of training

All subjects returned their fully completed training records allowing the calculation of average load and total volume load over the training period. In line with the intended design of the regimens the STR (85.41 ± 0.44 %), CL-1 (85.34 ± 0.40 %) and CL-2 (90.97 ± 0.65 %) groups trained at a significantly (*p* = 0.032) higher percentage of their 1RM than the HYP group whilst the CL-2 group also trained at a significantly greater (*p* = 0.048) percentage of their 1RM than the STR and CL-1 groups. The total volume load (normalised to body mass) completed in the HYP group (494.84 ± 98.70 kg) was significantly higher than that completed in the STR (*p* = 0.030, 393.85 ± 40.60 kg) and CL-1 (*p* = 0.038, 393.43 ± 53.77 kg) group.

### Acute responses

#### Metabolic responses

**Fig. 3:**
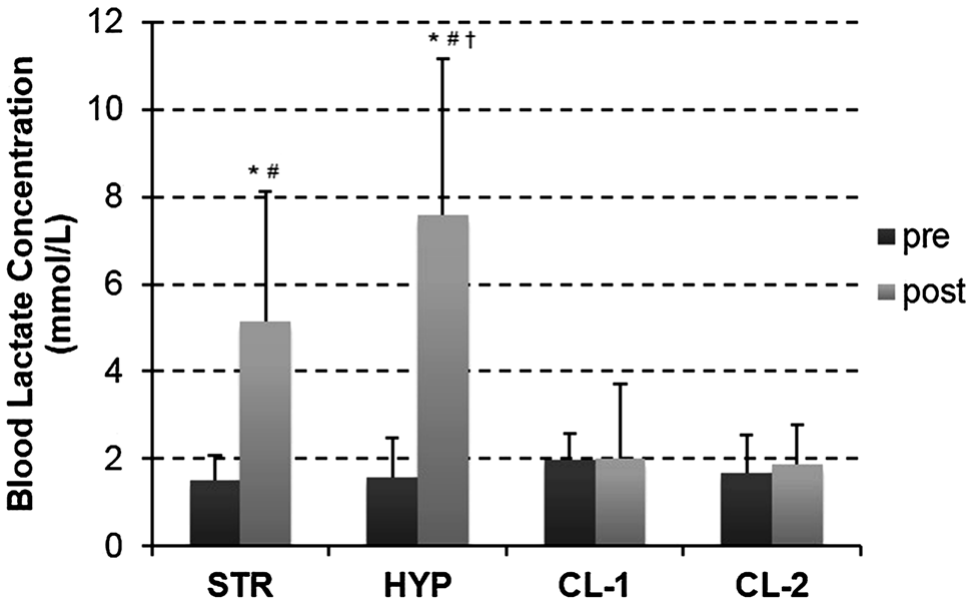
Pre- and post-workout values for blood lactate concentration (values are mean ± SD). *Asterisks* significant change from pre- to post-workout (*p* < 0.05), *number sign* significant difference from CL-1 and CL-2 (*p* < 0.01) and *dagger* significant difference from STR

Figure 3 shows the pre- and post-exercise values for BL concentration for the four groups where significant increases were observed only in the STR and HYP groups.

#### Repetition quality

The STR and HYP workouts elicited significant increases in the concentric TUT (16–21 %) and concentric impulse (11–17 %) associated with each repetition as the sets progressed (from the first to the last repetition). As shown in Table 4, the increases in TUT and impulse often proved significantly greater (*p* < 0.05) than those in the CL-1 and CL-2 workouts in many of the sets completed. The STR and HYP workouts elicited significant reductions (*p* < 0.05) in average concentric vertical force produced during each repetition within in all four sets (4–6 %). Furthermore, the reductions in vertical force for the STR and HYP workouts were significantly greater (*p* < 0.05) than for the CL-1 and CL-2 workouts during sets 1, 2 and 4. All four workouts elicited mean increases (2–49 %) in anterior force during each repetition as the set progressed (from the first to the last repetition) although the increases during the STR and HYP workouts were the only ones to reach statistical significance (*p* < 0.05). The STR and HYP workouts also elicited significant reductions (*p* < 0.05) in concentric velocity (22–46 %) and power (25–48 %) from the first to the last repetition of each set which were at times significantly greater (*p* < 0.05) than those reported for the CL-1 and CL-2 workouts (see Table 4).

**Table 4:** Percentage changes (mean ± SD) between the first and last repetition of each set for the key mechanical variables measured during the back-squat workouts for the four training groups

	Con. TUT (s)	Av. impulse (Ns/kg)	Average velocity (m/s)
Changes in mechanical responses (within-set)			
Set 1			
STR	↑19.97 ± 15.90*,^CL-1^	↑14.16 ± 14.92*	↓31.55 ± 26.61*
HYP	↑16.26 ± 12.30*	↑11.02 ± 12.36	↓21.52 ± 15.15*
CL-1	↑2.47 ± 8.54	↑1.04 ± 6.80	↓8.31 ± 14.39
CL-2	↑11.00 ± 15.89*	↑10.82 ± 16.19*	↓15.41 ± 21.65
Set 2			
STR	↑23.35 ± 15.55*,^CL-1,CL-2^	↑17.22 ± 15.00*,^CL-1^	↓43.42 ± 23.01*,^CL-1,CL-2^
HYP	↑20.32 ± 22.79*,^CL-1^	↑15.37 ± 22.68*	↓24.77 ± 18.26*
CL-1	↑1.10 ± 10.20	↓0.01 ± 10.09	↓4.14 ± 8.61
CL-2	↑4.52 ± 13.06	↑2.59 ± 10.80	↓8.56 ± 11.01
Set 3			
STR	↑18.95 ± 16.75*	↑13.11 ± 14.83*	↓45.83 ± 22.65*,^CL-1^
HYP	↑20.60 ± 15.25*,^CL-1^	↑14.75 ± 13.09*	↓28.89 ± 20.02*
CL-1	↑3.70 ± 9.12	↑7.28 ± 12.66	↓13.89 ± 21.23^STR^
CL-2	↑8.95 ± 14.03	↑ 5.86 ± 11.03	↓19.68 ± 28.55
Set 4			
STR	↑19.71 ± 14.99*	↑12.60 ± 16.40	↓40.40 ± 16.79*,^CL-1^
HYP	↑21.34 ± 14.99*,^CL-1^	↑13.48 ± 12.29	↓33.78 ± 23.62*
CL-1	↑5.72 ± 7.87	↑3.17 ± 7.82	↓8.10 ± 15.28
CL-2	↑9.27 ± 15.37	↑6.38 ± 18.70	↓11.88 ± 14.22
Set 5			
HYP	↑33.08 ± 29.90	↑25.52 ± 24.95	↓37.55 ± 14.76

*CL-1* significantly greater than the CL-1 condition, *CL-2* significantly greater than CL-2 condition

* Significant change from first repetition of the set (*p* < 0.05)

Table 5 shows the mean mechanical values for all of the repetitions completed in each workout. When averaged over the entire workout the TUT associated with individual repetitions in the CL-2 workout proved significantly longer than that attained during repetitions in the HYP (*p* = 0.030) and CL-1 (*p* = 0.042) workouts but not the STR workout. The repetitions in the STR, CL-1 and CL-2 workouts displayed a significantly greater level of force and impulse generation than the HYP workout (*p* < 0.05) with the impulse in the CL-2 workout also being greater than that of the CL-1 workout (*p* = 0.045). The repetitions in the CL-2 workout showed a significantly lower average velocity than the CL-1 workout (*p* = 0.042) and a significantly lower power output than the HYP (*p* = 0.035) and CL-1 (*p* = 0.042) workouts.

**Table 5:** Mean values for the mechanical variables measured during individual repetitions (averaged over the entire training session) for the four conditions

	Con. TUT (s)	Av. force (N/kg)	Av. impulse (Ns/kg)	Av. velocity (m/s)	Av. power (W/kg)
Mechanical characteristics—repetition average					
STR	1.53 ± 0.29	24.37 ± 1.92^HYP^	37.45 ± 9.04^HYP^	0.32 ± 0.12	7.56 ± 2.91
HYP	1.29 ± 0.15^CL-2^	20.66 ± 2.86	26.28 ± 3.91	0.40 ± 0.08	8.43 ± 1.72^CL-2^
CL-1	1.42 ± 0.22^CL-2^	24.45 ± 1.87^HYP^	34.79 ± 6.71^HYP,CL-2^	0.39 ± 0.10^CL-2^	9.51 ± 2.32^CL-2^
CL-2	1.75 ± 0.33	26.35 ± 1.84^HYP^	45.17 ± 4.56^HYP, CL-1^	0.25 ± 0.08	6.38 ± 2.03

*STR* significantly greater than STR, *HYP* significantly greater than HYP, *CL-1* significantly greater than CL-1, *CL-2* significantly greater than CL-2

## Discussion

This is the first study designed to investigate the chronic responses to traditional STR and HYP regimens and CL regimens which have intended to elevate repetition velocity and volume load. Our findings demonstrate that both types of CL regimen do not offer clear benefits for the development of maximal dynamic strength over STR regimens following a 6-week training period. The STR and higher volume load CL regimen did, however, elicit significantly greater improvements in maximal strength than the HYP regimen. From a scientific perspective, the findings enhance the understanding of the mechanical stimuli underlying strength adaptations indicating that the superiority of the STR and CL-2 regimens may have been associated with the optimisation of concentric impulse and TUT within each repetition performed during RT. In contrast, the smaller improvements demonstrated by the HYP and CL-1 regimens underlines that metabolic stress and repetition velocity are of secondary importance for the development of maximal strength.

The significantly greater improvements in 1RM for the STR and CL-2 regimens when compared to the HYP regimen are consistent with previous studies (Campos et al. 2002) which support the superiority of high-versus moderate-load regimens. In addition, the elevation of total resting time offered no significant benefits for maximal strength development which is consistent with the balance of previous research (Folland et al. 2002; Hansen et al. 2011). Although no significant differences were observed between the STR and CL-1 regimens in 1RM, the ES in STR (1.161) was larger than the ES in CL-1 (0.588). Whilst this observation is somewhat consistent with previous studies that have compared traditional and CL regimens equated by volume load (Goto et al. 2005), one of the key findings of the present investigation is that CL regimens which permit higher volume loads offer no benefits over STR regimens for the development of maximal strength. This implies that elevations in training-session duration (using CL workouts) may not be worthwhile when designing strength training programmes.

In line with the intended design of the workouts, metabolic stress was manipulated on a continuum type basis with the HYP workout resulting in the largest post-workout increases in BL concentration, the STR workout resulting in a smaller yet significant increase whilst the CL allowed the complete removal of any metabolic stimulus even when volume load was elevated. Enhanced metabolic accumulation as indicated by increases in BL concentration following the STR (244.84 %) and HYP (422.88 %) workouts is consistent with previous investigations (Kraemer et al. 1990; Nicholson et al. 2014). The present findings are in contrast to previous research (Denton and Cronin 2006) which reported significant increases in BL concentration following CL workouts. The heightened metabolic responses in the research most likely resulted from the equation of resting time between the regimens and the inclusion of less frequent rest intervals in the CL regimens which is often overlooked when interpreting the previous literature. From a scientific perspective, this is one of few studies that provide information regarding the relative importance of metabolic stress using exercises and workouts that are commonly used in training practice. Importantly, the post-training improvements for the CL groups suggest that metabolic stress is not the primary mechanism underpinning maximal strength adaptations. Whilst this finding is at odds with some previous research into blood-flow restriction (Takarada et al. 2002), questions still remain regarding the role of metabolic stress and circulating hormones in mediating muscle hypertrophy (West et al. 2009). Furthermore, evidence exists (Sakamaki et al. 2012) to suggest that light-load training with higher levels of metabolic stress may favour hypertrophy-specific strength gains which may not be desirable in sports where relative strength is important.

Since the large strength improvements in the CL-2 regimen suggest that metabolic stress is not paramount for maximal strength development, closer inspection of the acute mechanical characteristics may provide more information regarding the stimuli underlying strength adaptations. The mechanical measurements made during the four regimens demonstrated that the cumulative mechanical performance (over the course of a training session) was greatest during the HYP workout. Despite the fact that a greater cumulative performance during high-volume workouts has been previously used to explain studies which have not observed differences between high- and low-load regimens (Crewther et al. 2005), very few studies have examined acute mechanical performances alongside chronic changes in strength. The smaller 1RM improvements in the HYP regimen, therefore, provide important evidence against the importance of cumulative mechanical performance for maximal strength development. Instead, the greatest 1RM improvements occurred in the STR and CL-2 regimens which were characterised by greater peak mechanical responses, as has been previously suggested (Crewther et al. 2005). Crucially, the present findings, therefore, support the need to optimise the mechanics associated with each individual repetition when strength development is the training goal.

In terms of the importance of specific mechanical variables, it would appear that the CL-1 regimen was not optimally designed for strength development since the ability to offset fatigue-induced reductions in repetition velocity and power did not translate to greater strength improvements. In some respects, this observation supports the specificity of training adaptations (Kaneko et al. 1983) since maximal strength assessments are not typically associated with higher velocities and power outputs. Instead, it seems logical that the slower repetitions in the STR and CL-2 regimens closely resembled the 1RM assessment and contributed to the larger strength improvements in these regimens. More specifically, the higher load in the CL-2 workout and the fatigue-induced changes during the STR workout resulted in longer TUT and higher force outputs. The present findings actually support the product of these two variables (impulse) for strength development since impulse was greatest in the STR and CL-2 regimens. The notion that impulse, produced by elevating both TUT and force output and not by augmenting only one of the two variables (i.e. as in HYP regimens), may represent the critical stimulus underpinning strength development is consistent with previous investigations (Schuenke et al. 2012) which have demonstrated that the ability to maximise TUT may yield superior strength improvements as long as force output is not compromised. From a physiological perspective, it is possible that the higher impulse generation in the STR and CL-2 regimens may have resulted in the inhibition of force-feedback reflex mechanisms (McDonagh and Davies 1984) and/or an increased level of MU recruitment (Gordon et al. 2004). The stimulus-tension theory states that training loads need to be near-maximal and of sufficient duration if motor unit recruitment is to be maximised (Komi and Buskirk 1972); however, evidence exists on the contrary with similar neural responses being observed between different loading zones when the intensity of effort is maximised (Houtman et al. 2003; Carpinelli 2008). Whilst the present study does not provide firm evidence in support of any of the proposed mechanisms, the findings do support the positive effects of load elevation during CL regimens and the load–fatigue interaction during STR regimens to maximise impulse generation.

Although the dynamic strength improvements were also accompanied by mean increases in isometric strength measured during the isometric back-squat assessment, the improvements in isometric back-squat strength (5–9 %) were smaller than the 1RM increases (9–15 %) and no differences were observed between the regimens in the isometric strength improvements. This coheres with prior research (Coyle et al. 1981) which suggests that for a valid assessment of strength gain from a resistance training regimen, the testing modality should closely resemble the training conditions (i.e. type of muscle action, etc.). Given that the isometric back-squat protocol previously demonstrated high between-session reproducibility and significant correlations with the 1RM back squat (Nicholson and Bissas 2015), this highlights the need for coaches to closely consider the sensitivity of multi-joint isometric assessments for monitoring dynamic training improvements.

Metabolite accumulation has been proposed to mediate muscle hypertrophy via a range of mechanisms and although different levels of metabolic stress were observed, no measure of muscle hypertrophy was included in the present study. Importantly, however, the increases in isometric strength were accompanied by increases in sEMG of the key leg muscles along with significant main effects of time. Increases in sEMG activity have been widely used in support of neural contributions to increases in force output following a period of RT (Aagaard et al. 2000). It is important to consider, however, that increases in sEMG activity may not be entirely attributable to neural factors because a number of non-neural factors (e.g. blood flow, subcutaneous tissue) are also known to influence the recorded signal (De Luca 1997). Furthermore, the limitations of this technique do not allow specific changes (e.g. increased MU recruitment/firing rate) to be identified. Whilst the increases in neural drive are consistent with research which supports neural contributions during the initial stages of training (Chilibeck et al. 1998), the study provides further support to previous research (Hakkinen et al. 1987) which has highlighted a possible role for neural adaptations beyond the initial weeks of training. Whilst the findings are in agreement with previous studies that have observed increases in sEMG following heavy RT (Aagaard et al. 2000), there was no evidence to suggest that the STR or CL regimens resulted in greater neural stimuli despite the fact that the elevation of velocity (Linnamo et al. 2000) and load (Hakkinen et al. 1987) has been previously linked to increased neural contributions. In this respect, the present findings are in agreement with previous research (Iglesias-Soler et al. 2015) which has not observed differences in neural adaptations between traditional and CL regimens even using more clinical techniques (e.g. Interpolated Twitch Technique).

Despite the relatively short duration of the training intervention, the strength gains were transferred to explosive performance improvements as demonstrated by increases in isometric RFD, isokinetic peak torque and vertical jump height which are consistent with some previous investigations (Folland et al. 2002; Cormie et al. 2010). The fact that the increases in isokinetic peak torque at 30^°^/s were confined to the STR, CL-1 and CL-2 regimens was expected (Coyle et al. 1981) since the aforementioned regimens involved higher loads and slower velocities than the HYP regimen. Limited additional support for the concept of velocity specificity can be gained; however, no differences were observed in the magnitude of the improvements in isokinetic peak torque measured at 180^°^/s, isometric RFD or jump performance despite the HYP and CL-1 workouts emphasising repetition velocity to a greater extent than the STR and CL-2 regimens. In this respect, the findings are not at odds with the theory that it is the intention to move a resistance as fast as possible that is the key stimulus for improvements in explosive performance (Behm and Sale 1993). The lack of support for the concept of velocity specificity perhaps results from the use of the back squat as the training exercise, the examination of a relatively short training period or the failure to include a low-load ballistic training group.

Based on the present findings, it would appear that CL regimens do not offer clear benefits over STR regimens for the development of maximal strength although there exists the possibility that differences may have been observed if a longer block of training (e.g. >2 months) had been examined. The present findings, however, provide a valuable insight into the adaptations resulting from short blocks of training (6–8 weeks) that are commonly employed by coaches. From a practitioner’s perspective, it was interesting to note that the CL-1 regimen was associated with a significantly lower average RPE over the training intervention than the HYP regimen. Whilst some authors have concluded that perceived exertion is unaffected by rest interval length (Pincivero et al. 1999), the findings are consistent with research (Hardee et al. 2012) which supports lower RPE values when RT is performed with intra-set rest intervals. In this respect, the findings suggest that CL regimens provide an effective means of increasing maximum strength with lower levels of perceived exertion which may have implications for training adherence, motivation and the avoidance of overtraining. Interestingly, participants in the CL-1 regimen reported that the intra-set rest intervals initially enabled them to focus on the ‘quality’ of their back-squat repetitions, which is supported by the acute kinematic performance. On the contrary, it is important to note that the monotony of the CL regimens also negatively impacted on participants’ motivation at times and that the RPE and RPS of the CL-2 regimen increased sharply after the mid-point of training. Although more research is needed which includes more scientific markers (e.g. salivary IgA levels), the findings provide some evidence to suggest that CL regimens may be more suited to shorter, more targeted periods of training (<3 weeks).

In conclusion, CL regimens did not provide a more effective alternative to STR regimens for the development of maximal strength, explosive performance or the level of neural adaptations. Importantly, however, CL regimens were effective at reducing the metabolic demands of RT, limiting fatigue-induced reductions in repetition mechanics and allowed the utilisation of load-volume combinations that do not conform to the typical repetition maximum continuum (Baechle and Earle 2008). Based on these findings coaches should be mindful that CL regimens may result in contrasting acute responses to traditional STR regimens and as a result, rest interval frequency should be considered alongside rest interval duration when designing strength training interventions. From a scientific perspective, it would appear that the optimisation of repetition kinematics does not lead to greater strength adaptations but may be effective at reducing the level of perceived exertion. Instead, the use of STR regimens or CL regimens which elevate training load would seem to elevate the kinetic responses (i.e. impulse) that are central to the strength training stimulus. Furthermore, the optimisation of metabolic stress did not seem paramount to the strength improvements observed which is contrary to some recent suggestions. The findings highlight that intra-set as well as inter-set rest intervals are key considerations when designing resistance training regimens aimed at developing maximal strength.

## Abbreviations

ANOVA: Analysis of variance
Av: Average
BL: Blood lactate
CL: Cluster type
Con: Concentric
ES: Effect size
Ext: Extensor
Flex: Flexor
GM: Gluteus maximus
HYP: Hypertrophy type
MBIF: Maximal bilateral isometric force
MF: Median frequency
PT: Peak torque
RF: Rectus femoris
RFD: Rate of force development
RMS: Root mean square
RPE: Rating of perceived exertion
RPS: Rating of perceived soreness
RT: Resistance training
SD: Standard deviation
sEMG: Surface electromyography
STR: Strength type
TUT: Time under tension
VL: Vastus lateralis
1RM: One repetition maximum
